# A non-parametric framework for estimating threshold limit values

**DOI:** 10.1186/1471-2288-5-36

**Published:** 2005-11-07

**Authors:** Georgia Salanti, Kurt Ulm

**Affiliations:** 1 MRC Biostatistics Unit, Cambridge, UK; 2 Institute for Medical Statistics and Epidemiology, Technical University of Munich, Germany

## Abstract

**Background:**

To estimate a threshold limit value for a compound known to have harmful health effects, an 'elbow' threshold model is usually applied. We are interested on non-parametric flexible alternatives.

**Methods:**

We describe how a step function model fitted by isotonic regression can be used to estimate threshold limit values. This method returns a set of candidate locations, and we discuss two algorithms to select the threshold among them: the reduced isotonic regression and an algorithm considering the closed family of hypotheses. We assess the performance of these two alternative approaches under different scenarios in a simulation study. We illustrate the framework by analysing the data from a study conducted by the German Research Foundation aiming to set a threshold limit value in the exposure to total dust at workplace, as a causal agent for developing chronic bronchitis.

**Results:**

In the paper we demonstrate the use and the properties of the proposed methodology along with the results from an application. The method appears to detect the threshold with satisfactory success. However, its performance can be compromised by the low power to reject the constant risk assumption when the true dose-response relationship is weak.

**Conclusion:**

The estimation of thresholds based on isotonic framework is conceptually simple and sufficiently powerful. Given that in threshold value estimation context there is not a gold standard method, the proposed model provides a useful non-parametric alternative to the standard approaches and can corroborate or challenge their findings.

## Background

Estimation of a threshold limit value (TLV) is an important task in many medical areas, where risk factors are often scrutinized for values beyond which important medical or political decisions need to be taken – *e.g. *beyond which blood pressure value one should prescribe antihypertensive. The classical approach suggests several steps; a dose-response relationship needs to be established by applying a test for trend and then a set of candidate threshold values *x*_*i *_is identified. Finally, a threshold model is fitted at each of these candidate values. Considering an exposure variable in N doses *x*_*1*_,*x*_*2*_,...,*x*_*N*_, a widely applied threshold model for a binary outcome *p(x_*i*_) *is



where *f *is the identity function for the 'elbow' shape or the logit function for the logistic version, and *l *is the baseline risk. Then, the threshold is estimated as the exposure *x*_*i *_associated with 'better model' in terms of a goodness of fit criterion, *e.g. *the likelihood ratio statistics or the Akaike's information criterion. Usually only a set of plausible threshold locations is considered. To determine the set of candidate threshold locations, one has to screen visually the dose-response regression line and take a neighbourhood around a point where a 'jump' in the risk seems to occur. This practice, although widely applied in practice, is prone to bias and does not control efficiently the type I error.

A justified approach is to consider all doses *x*_*i *_as possible thresholds. The model

*f(p(x_*i*_) *= *l *+ *b *log *(x_*i*_/t)) if x*_*i *_>*t *    (2)

has been previously proposed together with a likelihood maximisation method for estimate the threshold and its confidence interval [1]. Modifications and further developments on such threshold value models can be found in [2,3].

A useful alternative to these parametric approaches is provided by isotonic regression. The main advantage compared to the models developed so far is that no specific assumption is made regarding the shape of the regression and a flexible step function is fitted. Moreover, the fitting algorithm automatically selects a small set of candidate threshold values without any a priori information about their location.

An underlying assumption throughout this paper is that a threshold value exists in every dose-response relationship. Evaluating this assumption is rather controversial. We align ourselves with those arguing that the question whether a threshold exists or not cannot be answered by means of statistical analysis [1,4]. Assuming a threshold is plausible in many toxicological and clinical studies, and even in cases where there is no biological justification, the threshold assumption can have practical implications.

Under this scope, we constrain this paper on presenting a framework for estimating the TLV given that its existence has been established as plausible. We explain the use of isotonic regression and we discuss how to select the actual threshold among the candidate locations suggested by the isotonic transformation.

## Methods

### Threshold estimation and isotonic regression

Isotonic regression is a maximum likelihood estimator under the assumption of a monotone dose response relationship. Whereas several algorithms are possible to fit the data, we use the Pooled Adjacent Violators Algorithm (PAVA) as it is the most efficient and comprehensive (see appendix) [5]. The result is a step function that summarizes the exposure in *L *constant risk groups (level sets) that are automatically selected by the algorithm without any a priori information about the location of the changepoints (here called isotonic cutpoints). The isotonic model can be thought of as a categorisation's procedure, where a set of cutpoints for the exposure variable is estimated with respect to the monotonicity in the associated risk [6].

Based on isotonic regression, we propose a two-stages approach, where all possible *x*_*i *_doses are evaluated. In the first stage, we take advantage of the fact that monotonicity is a pre-requisite condition for the threshold hypothesis. At this step, PAVA is screening all *x*_*i *_for compliance with monotonicity and re-estimates the regression shape under this condition. This screening is very powerful, given that isotonic regression provides a powerful and robust test for trend. Variations of the isotonic test and description of its advantages have been outlined in several papers [7-10]. The result is a step function with few cutpoints.

Once isotonic regression is fitted, the second step needs to be taken; the actual threshold needs to be selected among the isotonic cutpoints. On this purpose, we propose two methods described in the following sections.

The framework based on isotonic regression is in a sense equivalent to the methods that check all the dose values; however in isotonic regression most of the dose values are 'rejected' during the first stage on the basis of their compliance to the hypothesis of monotonicity, and only few are tested under the threshold value location hypothesis. An assumption underlying the proposed methodology is that the observations can be grouped in constant-risk exposure intervals. Whereas we acknowledge that this assumption may be questionable depending on the nature of the exposure, it is true for many environmental and toxicological factors.

### Selecting the threshold

#### Reduced isotonic regression

The cutpoints resulting from PAVA are estimated so that monotonicity is achieved and they do not necessarily correspond with a significant increase in the risk. The model can become more parsimonious if these 'non significant' level sets are eliminated. This parsimonious version is called reduced isotonic regression (RIR) and has been described and studied elsewhere [6,11]. When the outcome is binary, the reduction is accomplished by a sequence of Fisher tests for the adjacent 2 × 2 tables. The correction for multiple comparisons is made by a-priori estimation of the elimination significance level ε* in a permutation procedure so that the actual type I error is kept constant at *a *(e.g. *a *= 5%). The result is a step function with less level sets than the original isotonic model. Moreover, each cutpoint defines now a significant increase in the associated risk. Consequently the first 'step' in an RIR line will indicate the TLV. The model can be thought of as an extension of the 'elbow' threshold model in equation (1) where the first level set estimates the background risk *l *and the first cutpoint defines the threshold *t*.

#### Closed testing procedure

An alternative for selecting the threshold out of several candidate locations suggested by PAVA is to consider the closed family of hypotheses. The classical closed testing approach suggests that a hypothesis *H *is tested only if all hypotheses that contain *H *have been rejected at some fraction of the significance level *a*. The family-wise error is controlled, but power is usually low.

An option to increase the power is to make one part of the regression line conditional to the other [12]. This concept suits the TLV estimation context where the beginning of the dose-response is more important than its end and testing for threshold among levels of higher exposure is conditional on the rejection for the lower exposure levels.

**Figure 1:**
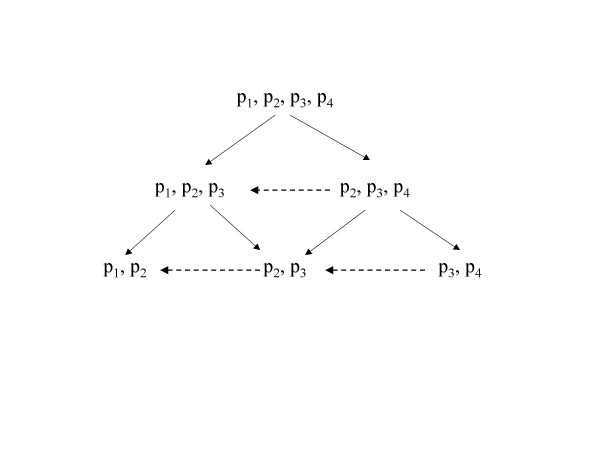
**Closed testing procedure. **The arrows show the direction of the conditional testing.

The diagram in figure 1 presents a closed family of hypotheses. Every 'vertical' hypothesis about testing an isotonic cutpoint *x*_*i *_is conditional on the rejection of the hypothesis above (vertical conditioning) and testing every hypothesis on the right hand side is conditional on the retention of the hypothesis on its left (horizontal conditioning).

1. Every hypothesis *H(l,k):' the risk is constant between two isotonic cutpoints x*_*l *_*and x*_*k*_*' *and its nested hypotheses are conditional on the rejection of every hypothesis *H(l',k') *with *l< l' *and *k< k'*

2. Retain every hypothesis implied by any other hypothesis that has not been rejected

Consider for example that isotonic regression suggests 4 isotonic cutpoints as candidate thresholds defining 4 level sets with associated risks *p*_*1*_*,...,p*_*4 *_(figure 1). The direction of the arrows shows the conditioning in testing. The procedure starts by evaluating *H(1,3) *(between *x*_*1 *_and *x*_*3 *_the risk is constant, *i.e. p*_*1 *_= *p*_*2 *_= *p*_*3*_). If we retain, then we test *H(2,4) *(rejection: threshold = *x*_*3*_, no-rejection: no dose-response relationship). If we reject *H(1,3) *then we test *H(1,2) *(rejection: threshold = *x*_*1*_, no-rejection: continue by testing *H(2,3)*). In every step, the exact isotonic test for trend is used [10].

When the conditioning occurs 'horizontally' in addition to the vertical restriction as in figure 1 (dotted arrows), then the power increases. Consider that *H(1,4) *is not true due to *H(1,2) *being not true. Then with only vertical restriction the probability to correctly reject *H(1,2) *using *α *= 5% is 0.95^5^+0.95^3^0.05^2^+0.95^2^0.05+0.95^4^0.05 = 0.862 whereas with the horizontal conditioning it is 0.95^2^+0.95^2^0.05 = 0.947.

Compared to RIR, this approach is easier to apply, but provides no information about the shape of the dose-response relationship after the threshold. Closed testing elimination concentrates on increases in the risk whereas RIR achieves a complete re-estimation of the regression line with overall improvement in the fit. For both methods, bootstrap can be used to calculate the confidence intervals.

### Extension: The isotonic surfaces model

There are situations where thresholds need to be identified for multiple factors that interact. One of the main advantages of the presented methods is that they can be easily combined with multivariate isotonic models; either the isotonic-surfaces model where the level sets correspond to combinations of the predictor variables or the additive isotonic model [6,13,14].

Consider two continuous predictors *x *and *y*, a binary outcome and their three-dimensional scatter plot. The isotonic surfaces model is simply a surface fitted in the scatter plot that is monotone along both *x*- and *y*-axes. Two-dimensional blocks of constant response are built, and a reducing procedure similar to the one followed in univariate regression is applied to improve parsimony. Details on fitting and elimination algorithms can be found in [6]. This model can be used to estimate two-dimensional thresholds, as we exemplify in the application.

### Simulation study

The performance of the two methods described above as threshold detectors depends upon their power to establish a dose-response relationship. Consequently, in this simulation study we first concentrate on evaluating the power to reject the constant risk assumption. Higher power is associated here with greater proportion of non-constant estimated regression lines. Subsequently we evaluate the ability to detect the isotonic level set that is associated with a threshold, conditional to the rejection of the constant risk assumption.

**Figure 2:**
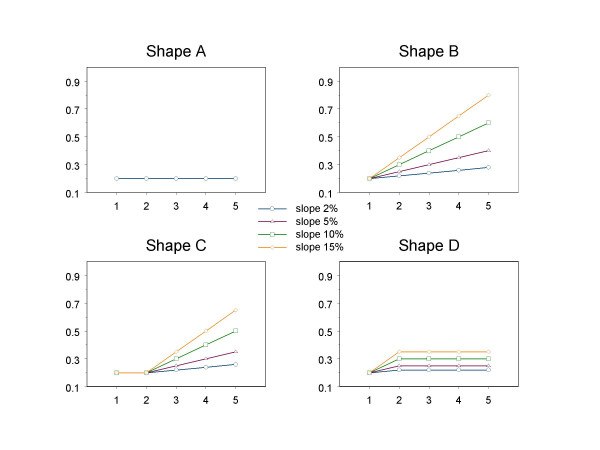
Regression shapes studied in the simulation study.

We simulate under 5 isotonic level sets, with equal number of observations per group that varies from 50 to 250. Four shapes for the isotonic groups are studied. For each combination of slope (for the increasing part of the regression) and sample size, 1000 simulations are analyzed (figure 2).

Shape A assumes no dose-response and the type I error of the methods as 'tests for trend' can be accessed. Shape B corresponds to a linearly increasing relationship starting with 20% risk and a threshold is assumed at the first dose group. Shape C represents a segmented regression line assuming a threshold at the second dose level. The baseline constant risk is 20% and afterwards the risk increases linearly. The last type of regression (Shape D) is also a segmented line but assumes a threshold at the first cutpoint. Between the first two dose groups the risk increases linearly and is flat between the second and the fifth. This dose-response relationship is close to the horizontal line (figure 2). In every shape that includes linear regression part we studied different slopes of 2%, 5%, 10% and 15%.

### Application to MAK study

The study "Maximale Arbeitsplatz-Konzentration" was conducted by the German Research Foundation and comprises among others a cohort of 920 smoking workers exposed to a mixture of dust, mainly from iron, steel, foundry and engineering [15,16]. The endpoint of the study was chronic bronchitic reaction (CBR). Dust concentration and the length of exposure have been previously established as important risk factors. The goal in this present application is first to estimate a TLV for the cumulative exposure in dust over time in a univariate regression and subsequently to assess a two-dimensional threshold for both dust concentration and duration of exposure.

## Results

### Results from simulations

#### Establishing dose-response relationship

When RIR is applied, a dose-response relationship is established for shape A in 5% of the simulated samples. This was somewhat expected, since the elimination procedure is designed to keep type I error fixed at the nominal level [6]. The closed testing approach yields an error rate of 2%.

Table 1 presents the estimated power to establish a dose-response relationship in shapes B-D for sample size 100 and the two lower slopes. For comparability between the two methods, the calculations were carried out by calibrating ε* so that the error rate is 2% for RIR. The RIR performs better for shapes B and C, and although for flat regression lines is not very powerful, its power increases very fast with higher slopes or sample size. The power lies between 78% and 99% for slopes higher than 5% for every sample size. For shape D, both methods are largely underpowered with closed testing procedure having double the power of RIR. In this regression line, the contrast in the risk is between a single level set versus four levels having higher but equal risk, and both elimination procedures are likely to miss it and pool all level sets together. For RIR sample size as high as 200 observations per level set and a slope higher than 10% are required to achieve power of at least 65%.

**Table 1:** The average power for reduced isotonic regression and closed testing procedure in establishing a dose-response relationship for the two lower slopes and sample size 100 observations per level set.

**Shape**	**Slope**	**Power to establish dose-response relationship**	
		
		**Closed testing**	**RIR**
B	2%	19%	27%
	5%	29%	88%
C	2%	10%	13%
	5%	32%	40%
D	2%	7%	2%
	5%	19%	10%

#### Assigning thresholds to the level sets

The assignment of thresholds was studied among the datasets where a dose-response relationship was established. Closed testing procedure was less successful than RIR for slopes lower than 5% and sample size less than 100 observations per isotonic level set. For these values, the estimated threshold in shapes B and D was higher than the first level set – and thus overestimated- in 99% of the cases. It is only after sample size 150 that closed testing procedure starts getting a bit more successful, following similar patterns as the ones observed for RIR.

Table 2 presents the threshold assignment for RIR. The estimated thresholds in shape B do not seem to follow any specific pattern for slope 2% where every cutpoint has more or less the same probabilities to be selected as threshold. However, for slopes higher than 5% or greater sample size, the first group is most likely to contain the threshold.

**Table 2:** Average probability (for sample size and slope) to select a threshold at a given isotonic cutpoint applying RIR, average for sample size and slope. In italic appears the number that is considered to be the pertinent estimation in each shape.

	**Probability to select an isotonic cutpoint as threshold among the non-constant lines**			
**Shape**	**1st**	**2**^nd^	**3rd**	**4th**

B	*47%*	37%	14%	3%
C	4%	*68%*	24%	5%
D	*96%*	3%	1%	0.5%

**Figure 3:**
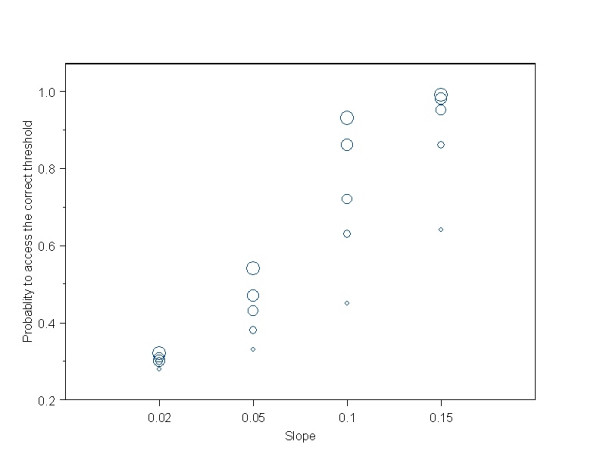
**Power of reduced isotonic regression to detect the correct threshold in shape C for different slopes. **The size of the circle is proportional to the sample size per dose group (N = 50, 100, 150, 200, 250).

Shape C is of particular interest regarding detection of the threshold location. The probability to assess it correctly, averaged for sample size and slope was 68%. Figure 3 presents the power in greater details for every slope and sample size. The ability to detect the threshold increases sharply with the slope and sample size in an almost linear way. It is remarkable that the probability to assess a threshold at the first level set is very low (4%) and decreases with sample size. Both methods present an important tendency to assign thresholds to the adjacent group that corresponds to a higher dose is observed (average probability 24%). The estimated background risk is slightly biased and lies within 20–20.6%. When the true threshold value was assumed to be at the third dose level, we did not observe any important differences in the performance of both methods apart from a slight drop in the already low probability to assign a threshold to the first cutpoint.

In shape D, the first group has the greatest probability to be selected as threshold, which is in agreement with the underlying regression shape.

### Application to CBR study

**Figure 4:**
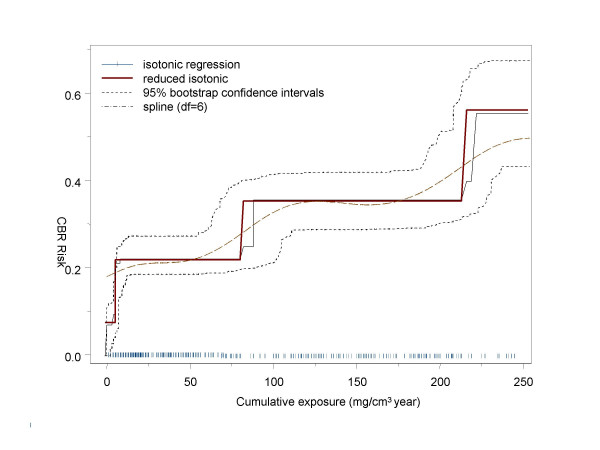
**Full isotonic and reduced isotonic regression fitted in the sample from Munich. **The 95% confidence bands correspond to the reduced regression.

Figure 4 depicts the fitted isotonic regression with 11 dose groups along with its reduced version (4 dose-groups) truncated for clarity at 250 mg/m^3^year. A smoothing spline with 6 degrees of freedom shows that the level sets defined by RIR are reasonable. The threshold was estimated at 7 mg/m^3^year with 95% bootstrap confidence intervals (4.9, 10) mg/m^3^year and background risk of 7.6%.

The isotonic level sets were analyzed with the closed testing algorithm. The hypothesis H(1,10) for equal risk between the lowest exposure and the tenth cutpoint (exposure at 390 mg/m^3^year) was rejected with exact p-value < 0.001 and so did all nested hypotheses up to H(1,4). Hypothesis H(1,3) was retained (p-value = 0.33). This means that the first three level sets are lumped together, and the third isotonic cutpoint (7 (5–9.9) mg/m^3^year) defines the threshold. The logistic regression (equation (2)) estimated in a threshold of 7 (5–8) mg/m^3 ^with a likelihood ratio statistics of 7.3 compared to the model with no threshold (p-value < 0.01).

**Figure 5:**
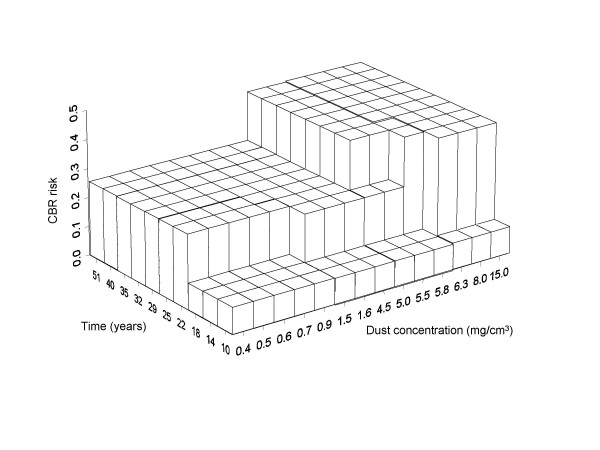
**Two-dimensional reduced isotonic regression modelling the dust concentration and the duration of exposure. **The labels in the bars are the upper limit for the time and concentration intervals.

In figure 5 we present the two dimensional isotonic regression, after the reducing procedure. Three major blocks are built for dust exposure and duration, and the first important step sets the threshold for dust at 4.5 (3.1, 7.2) mg/m^3 ^and more than 18 years of exposure. This estimation is compatible with the threshold from the extended logistic model in equation (2) using time and *ln*(dust concentration) as covariates (3.8 [1.4, 4.6] mg/m^3^). However, the isotonic surfaces model gives more detailed information according to time subgroups. For less than 10 years exposure for example, no increase in the risk is observed.

## Discussion

In this paper we presented a method for estimating thresholds that does not rely on any parametric assumption. Isotonic regression is flexible and easy to apply, and detects thresholds with relative good power. It can be applied both with continuous exposure variables and categorical, where every dose level is actually a range of values. In both cases the same framework is followed, since isotonic regression groups the continuous predictor in constant risk level sets, as we exemplified in the application section. At this point some limitations of the methods should be discussed. Whereas we believe that the bootstrap confidence intervals offer good coverage for the true threshold, the point estimate may be biased. Some observations right after the threshold may have lower risk due to sampling error, misclassification or different individual response, and subsequently PAVA will pool them all together shifting the isotonic cutpoint towards higher exposures. This means, the threshold may be overestimated, as the simulation study revealed. Assigning a threshold to the whole level set rather than its upper cutpoint may be a reasonable compromise. When the exposure is in continuous form, the researcher has the advantage of more detailed investigation; the level set that appears to have the threshold can be re-analysed using non-isotonic models to reveal any particularities of the data. However the practice will be suggested by the working definition of the threshold *i.e. *whether a 'No Observed Adverse Effect Level' or a 'Lowest Observed Adverse Effect Level' is of interest.

Modifications of the proposed models are possible. Hothorn suggests a procedure based on odds ratios [17]. He argues that when one wants to detect an important increase in the risk, the use of confidence intervals is more accurate than comparing p-values. This idea can lead to a modification of backward elimination; instead of using p-values, the confidence intervals for the odd ratios could indicate a significant increase in the risk.

To our knowledge, there is no statistical model for threshold estimation that claims high power. Thus, it is important that more than one approach should be applied to confirm a TLV's location. External validation for the applied models can also provide useful information since there are cases where the data can be fitted by a variety of models (that consider a threshold or not) and all of them may fit well yielding however discrepant estimates or contradictory conclusions [1,18]. Modelling the data using smoothing splines or fractional polynomials would be useful in revealing the true shape of the relationship and avoid misinterpretations. In practice however the final decision about establishing a threshold is often taken on an ethical, political and economical basis. In threshold value estimation context statistical methods are tools that can eventually inform and direct the decision making process.

## Conclusion

If the dose-response relationship is flat (slope less than 5%), the closed testing procedure fails to reject the constant risk assumption and thus has little power to detect the correct threshold. The method based on RIR is preferable as more powerful in most of the studied cases. When the increase in the risk is sufficiently high (at least 5%) both elimination approaches will detect the threshold successfully with RIR presenting the best results. In such situations, the threshold value estimation based on isotonic framework is conceptually simple and powerful. Given that no threshold value estimation method has been proven to have high power, isotonic regression provides at least a useful non-parametric alternative to the standard approaches and can corroborate or challenge their findings.

## Appendix

### The pooled adjacent violators algorithm

Consider a set x_1_,x_2_,...,x_N _of dose groups in increasing order, the observed outcome g(x_i_) for each dose group and the weights w_i_. To estimate g*(x_i_) the isotonic regression of g(x_i_), the pooled adjacent violators algorithm outlined below is the most popular approach. Note that the isotonic estimator is a maximum likelihood estimator under the monotonicity assumption. For simplicity assume only non-decreasing trend.

If g(x_i_) is in non-decreasing order then g*(x_i_) = g(x_i_).

Otherwise there is somewhere a violator such that g(x_i_) > g(x_i+1_) for some x_i_. Replace these two values by their weighted average

Av(g(x_i_),g(x_*i*+1_)) = (w_i_g(x_i_) + w_i+1_g(x_i+1_))/(w_i _+ w_i+1_).

Now the elements x_i_, x_i+1 _form a block called level set (LS) or solution block. If the new set of N-1 values is isotonic, then g*(x_i_) = g*(x_i+1_) = Av(g(x_i_),g(x_i+1_)) for the violator and g*(x_i_) = g*(x_i+1_) for all other observations.

If the set is not isotonic repeat the procedure using the new set of values.

The algorithm assuming decreasing trend is similar. Starting from the end of the shape and proceeding backwards (reversing the monotonicity) would give the same results.

## Competing interests

The author(s) declare that they have no competing interests.

## Authors' contributions

The first author performed the analysis and the simulation study while both authors contributed to the writing.

## Pre-publication history

The pre-publication history for this paper can be accessed here:


